# Insights into Copy Number Variation Architecture in Black Bengal Goat Genome

**DOI:** 10.3390/ijms27094045

**Published:** 2026-04-30

**Authors:** Sonali Sonejita Nayak, Shikha Mittal, Manjit Panigrahi

**Affiliations:** 1 Division of Animal Genetics, Indian Veterinary Research Institute, Izatnagar, Bareilly 243122, UP, India; sonalisonejita@gmail.com; 2 Department of Biotechnology and Bioinformatics, Jaypee University of Information Technology, Waknaghat, Solan 173234, HP, India; shikha.mittal@juitsolan.in

**Keywords:** Black Bengal goat, copy number variation (CNV), fecundity, structural genomic diversity, skin quality, whole-genome sequencing

## Abstract

Copy number variations (CNVs) are a major source of structural genomic diversity that influences adaptation, reproduction, and production traits in livestock. The Black Bengal goat, an economically important Indian breed known for its high fecundity, superior skin quality, and resilience to humid tropical climates, was studied to uncover its structural genomic landscape. We performed whole-genome CNV analysis using high-depth (10×) sequencing data from eight individuals. A total of 31,816 copy number variants (CNVs) were identified, predominantly duplications, with an average length of approximately 45 kb. These CNVs were combined into 8910 copy number variation regions (CNVRs) covering approximately 0.15 Gb (about 5.3% of the autosomal genome). CNVR hotspots were mainly located on chromosome 1. Gene annotation showed that regions overlapping with CNVs and CNVRs contained more than 1987 protein-coding genes involved in pathways related to immunity, reproduction, metabolism, and extracellular matrix (ECM) organization. The presence of CNVs involving genes such as GDF9 and BMPR1B on chromosomes 7 & 6, respectively, is important because it indicates that the breed has a high reproductive capacity due to dosage-sensitive duplications. Changes in the extracellular matrix and increased dermal strength have been linked to duplications of genes such as COL6A1, LAMC2, LAMB3, FMN1, and CLDN1. This helps explain the superior hide quality of the breed. This research offers a comprehensive map of CNVs and CNVRs within the genome of the Black Bengal goat. It demonstrates how these duplications lead to structural changes that enhance both reproductive performance and skin resilience. These findings provide a valuable genomic resource for future marker-assisted selection, comparative genomics, and conservation breeding programs aimed at preserving indigenous goat populations.

## 1. Introduction

One of the most important ways that animals can pass on genetic diversity is through genomic structural variants, specifically copy number variations (CNVs). CNVs are DNA segments that are longer than 1 kb that differ in copy number between individuals.

CNVs can alter gene dosage, disrupt gene structure, or change regulatory landscapes, which can have significant effects on gene expression and phenotype. This differs from single-nucleotide polymorphisms (SNPs), which change only one base. In livestock, CNVs have been associated with immunity, fertility, pigmentation, and metabolic efficiency, augmenting SNP-based variation in the development of adaptive and production traits [1,2].

Recent genome-wide CNV studies have uncovered pronounced breed- and environment-specific structural patterns in domestic animals. For instance, CNVs in KIT and ASIP control the coat color in goats; duplications in PRLR and GHR affect lactation performance; and structural variants in BMP15 and BMPR1B affect litter size in small ruminants [3]. Certain genomic regions have been identified as harbouring CNVs linked to adaptation in comparative studies of goat populations worldwide. Since then, studies have shown that some goat breeds share distinct genetic characteristics. In Chinese goat breeds, study [4] discovered gene duplications linked to olfactory perception and immunological responses. Dong and colleagues found that in Cashmere and Boer goats, CNVs were associated with reproductive parameters and coat traits [5]. Jin and his team found that Tibetan goats’ adaptation to high elevations was associated with CNVs [6]. However, research on Indian Black Bengal goats is limited, resulting in a limited understanding of the structural genomic underpinnings of their exceptional adaptability and productivity. The Black Bengal goat is an important breed for the culture and economy of eastern India. It is known for maturing sexually early, having 2–3 kids per kidding, and being naturally resistant to disease when raised by smallholder farmers. The global leather industry values its fine-grained, high-tensile skin. These two traits—high fecundity and excellent skin quality—make the breed a great model for studying how structural genomic variation affects adaptive and productive traits [7]. This breed’s genome-scale structural organization has not been well understood because previous molecular research has primarily concentrated on mitochondrial diversity, microsatellite variance, or SNP genotyping.

CNVs provide a direct connection between an organism’s genotype and phenotype. Alterations in reproductive regulators such as Growth Differentiation Factor 9 (GDF9) and Bone Morphogenetic Protein Receptor Type 1B (BMPR1B) can modify TGF-β signaling, influencing the number of developing follicles and the ovulation rate [8]. CNVs in genes related to the extracellular matrix (ECM) and the basement membrane, such as COL6A1, LAMC2, and LAMB3, may also impact the production of collagen, the strength of the dermis, and the flexibility of tissues. These factors are closely related to the different skin types observed among breeds [9]. In addition, identifying recurrent CNV regions (CNVRs)—which are overlapping CNVs present in several individuals—can uncover signs of selection at the population level and highlight functionally important regions of the genome.

In this context, the current study represents the first high-resolution genome-wide examination of CNVs and CNVRs in the Black Bengal goat utilizing high-depth whole-genome sequencing (WGS). The specific objectives were to: (i) identify and characterize CNVs across the genome; (ii) define CNVRs and chromosomal hotspots of structural variation; (iii) annotate genes that overlap with CNVs and CNVRs, examining their functional contributions; and (iv) focus on structural variants in critical fecundity and skin-quality genes—GDF9, BMPR1B, COL6A1, LAMC2, LAMB3, and CLDN1.

This study clarifies the structural genomic factors that contribute to prolificacy, skin quality, and environmental resilience in the Black Bengal goat through the integration of read-depth-based CNV detection, CNVR merging, and functional annotation. The resulting CNV–CNVR atlas serves as a significant genomic resource for selection, conservation, and evolutionary research in indigenous Black Bengal goats of India.

## 2. Results

### 2.1. Genome-Wide CNVs and CNVR Landscape

The whole genomes of eight Black Bengal goats were sequenced, with each individual generating an average of 202 million paired-end reads, and over 94% of bases exceeding a Phred quality score of Q30. Genomic relatedness (PI_HAT) estimates were low (<0.05), confirming that the sampled individuals are not closely related. With a mean sequencing depth of 10×, we identified 31,816 high-confidence CNVs across all autosomes by aligning the sequencing reads to the *Capra hircus* ARS 1.2 reference genome. The CNVs ranged in size from 1.1 kb to 2.8 Mb (mean = 45.3 kb). From 39 Mb in sample 786 to 342 Mb in sample 796, the total CNV-affected genomic span per individual ranged from 1.3% to 11.4% of the autosomal genome (Table 1). Combining CNVs that overlapped between individuals resulted in the identification of 8910 CNVRs that covered roughly 0.158 Gb, or 5.3% of the reference genome. Of these, 6934 (77.8%) were shared by two or more individuals, while 1976 (22.2%) were unique to a single goat.

### 2.2. Chromosomal Distribution and CNVR Hotspots

**Figure 1:**
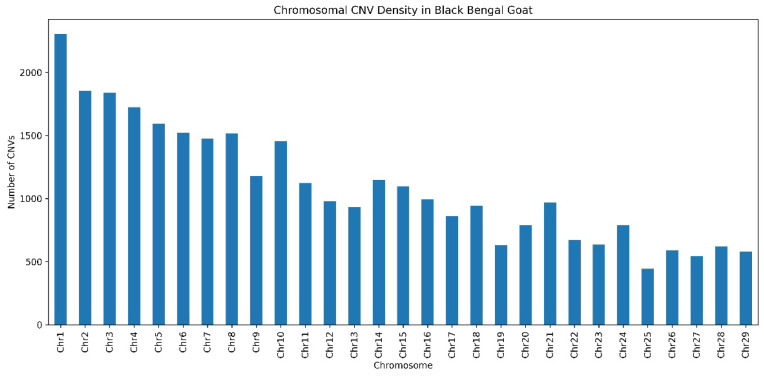
**Chromosomal distribution of copy number variations (CNVs) in Black Bengal goat.** The bar plot shows the number of CNVs detected across autosomes (Chr1–Chr29) of the Black Bengal goat genome. The x-axis represents individual chromosomes, while the y-axis indicates the total CNV count per chromosome. A marked enrichment of CNVs was observed on Chr1, identifying it as a major hotspot for structural variation, whereas the remaining chromosomes exhibited comparatively lower CNVs densities, highlighting the non-random genomic distribution of CNVs.

CNVs and CNVRs were unevenly distributed across the genome of the Black Bengal goat. The highest CNV densities were observed on chromosomes 1, 2, 3, and 4, which together accounted for roughly one-fourth of all detected CNVs (Figure 1). These chromosomes represent major CNV hotspot regions characterized by large, overlapping duplications (>100 kb) shared among multiple individuals. Notably, two key reproductive genes—BMPR1B (29.89–30.3 Mb) and GDF9 (66.023–66.028 Mb)—were located within CNVR-enriched segments. A detailed summary of CNVR-enriched regions and their associated genes is provided in Supplementary Table S1. The prevalence of such large, shared duplications suggests selective retention of dosage-sensitive loci associated with folliculogenesis and ovulation rate, contributing to the exceptional prolificacy of the Black Bengal breed. The genes COL6A1 and COL6A3, which aid in the production of collagen, were also found on chromosome 1. LAMC2 and LAMB3 are found in another hotspot on chromosome 16 (62.84.4–72.30 Mb) and are crucial for basement membrane adhesion. These CNVR-enriched areas most likely represent chromosomal clusters that have been selected due to characteristics that influence skin structural integrity and reproductive success. These characteristics are representative of the adaptability and reproductive performance of the Black Bengal goat. These traits may be typical of the Black Bengal goat’s ability to adapt and be prolific.

### 2.3. Inter-Individual Variation

**Figure 2:**
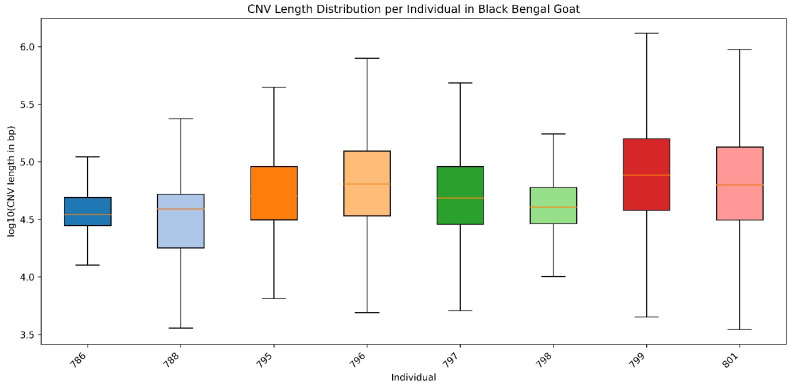
**Distribution of copy number variations (CNVs) lengths per individual in Black Bengal goat.** Colored boxplots represent the distribution of log10-transformed CNVs lengths (bp) detected in individual animals. Each box corresponds to one individual, with the median shown as a central line, the interquartile range (25th–75th percentile) as the box, and whiskers indicating the range excluding outliers.

The number of CNV occurrences per individual ranged from 290 to 7230, indicating significant structural variability among individuals. Samples 801 and 796 showed the most extensive duplication coverage (~12% of the genome), while sample 786 had the fewest CNVs (<2%) (Figure 2). The average ratio of duplications to deletions was approximately 15:1 and showed minimal variation across individuals. Most CNVs were less than 100 kb; however, samples 801 and 796 had substantial duplications (>500 kb) that often included multiple gene clusters. Most CNVRs were less than 50 kb, but many overlapped with genes that are associated with reproduction, production, and immune function.

### 2.4. Functional Annotation and Enrichment of CNV/CNVR Genes

Gene annotation revealed that 9157 unique protein-coding genes overlapped with CNVs and CNVRs, many of which were enriched in multiple biological processes (Supplementary Table S2). The biological processes and pathways associated with immune modulation, extracellular matrix (ECM) architecture, and reproductive physiology were significantly overrepresented in the Gene Ontology (GO) and KEGG pathway enrichment analyses (FDR < 0.05). Signal transduction (GO:0007165), extracellular matrix organization (GO:0030198), reproductive process (GO:0022414), and immune system process (GO:0002376) were among the most enriched GO terms. The top recurrent CNV-overlapping genes shared across multiple individuals are listed in Table 2, highlighting potential selection on reproduction, ECM organization, and immune signaling pathways. TGF-β signaling, ECM–receptor interaction, and cytokine–cytokine receptor interaction were also identified as significantly enriched KEGG pathways. All of these results highlight how CNV-associated structural variations influence gene networks that affect immunological responsiveness, dermal tissue architecture, and folliculogenesis—essential biological traits that support the Black Bengal goat’s adaptability and reproductive performance.

### 2.5. CNVRs Associated with Fecundity Genes

GDF9 and BMPR1B, two well-established fecundity genes, were shown to be present in shared CNVRs on chromosomes 7 & 6, respectively. Samples 795, 786, and 796 harbored duplications of the BMPR1B CNVR, which was 450 kb (29.89–30.34 Mb), while the GDF9 CNVR was 5 kb (66.023–66.028 Mb). Oocyte maturation and ovulation are critically regulated by GDF9 and BMPR1B, two essential elements of the TGF-β signaling pathway. The identified duplications in these genes may amplify gene-dosage effects because the breed is characterized by producing two to three offspring per kidding. This may provide molecular evidence that structural variation is associated with the higher fertility of Black Bengal goats.

### 2.6. CNVRs Associated with Skin and ECM Quality

Several CNVRs encompassed extracellular matrix (ECM) and skin-related genes involved in collagen synthesis, dermal adhesion, and follicular architecture. COL6A1 and COL6A3 (21 kb; samples 786, 795, 796) were linked to the assembly of collagen fibrils, LAMC2 and LAMB3 (70 kb & 42.5 kb; samples 795, 797) linked to the anchoring of basement membranes and FMN1 (437 kb; sample 795, 786) related to dermal cytoskeletal organization were among the notable duplications. These genes were associated through Gene Ontology clustering to “extracellular matrix organization” and “basement membrane assembly,” indicating their combined role in the breed’s high-tensile, fine-grained skin (Table 3). The remarkable skin texture and strength that have made the Black Bengal goat famous world-wide may be partly attributed to these CNVRs.

### 2.7. CNVR Sharing and Adaptive Modules

Of the 8910 CNVRs identified, 6934 (77.8%) were shared by ≥2 individuals, and 61 core CNVRs were shared by ≥4 individuals, representing population-level putatively adaptive regions. These shared CNVRs were overlapping GDF9, BMPR1B, COL6A1, LAMC2, and CLDN1, reflecting potentially selection-driven retention of reproductive and dermal adaptation modules. Such recurrent duplications may indicate a structural convergence mechanism that maintains key genes that enhance prolificacy and environmental resilience under tropical stressors.

### 2.8. Functional Integration of CNVR-Associated Genes

The four main functional classes of CNVR-associated genes were metabolic adaptability, immunological defense, skin and extracellular matrix (ECM) organization, and reproductive regulation. GDF9, BMPR1B, KITLG, ADAMTS1, and FGF5 belonged to the reproductive cluster and are involved in gonadal signaling, ovulation, and folliculogenesis. Genes involved in skin and ECM organization—such as COL6A1, LAMC2, LAMB3, CLDN1, and FMN1—are associated with collagen assembly, basement-membrane integrity, and dermal adhesion.

The immune defense group comprises CD80, IL10RB, ROBO1, and USP25, which participate in cytokine signaling and host–pathogen interactions. The metabolic adaptation cluster, including GBE1, TIAM1, and NXPE3, was enriched for pathways related to glycogen metabolism, cellular stress response, and energy homeostasis.

Interestingly, the co-localization of ECM-structural genes (COL6A1, LAMC2, and LAMB3) with fecundity-related genes (GDF9, BMPR1B) points to a pleiotropic adaptation process in which structural variants improve somatic resistance and reproductive efficiency at the same time (Table 3). The Black Bengal goat’s unique combination of high prolificacy and outstanding skin quality—qualities essential to its evolutionary fitness and economic significance— may be partly attributed to this genetic synergy.

## 3. Discussion

Copy number variations represent a major type of structural variation in the genome, resulting from the duplication or deletion of segments of DNA. They contribute to genetic diversity and phenotypic variation among organisms. CNVs are common in goat breeds worldwide, according to recent genome-wide analyses, highlighting their importance for features related to growth, reproductive performance, disease resistance, and adaptation. Genome-wide studies have identified thousands of CNVs per individual goat, covering between approximately 9% to 39% of the autosomal genome, depending on the breed and the sampling strategy used. For instance, the ADAPTmap Project analyzed 1023 samples from 50 goat breeds and identified 6286 putative CNVs merged into 978 CNV regions (CNVRs), which together span approximately 8.96% of the genome [10]. In a separate study, 32,711 autosomal copy number variation regions (CNVRs) were found in 11 Indian goat breeds. This showed notable genetic variation among the breeds and emphasized selection signatures associated with climate adaptation [11].

The prevalence and distribution of CNVs are dependent on breed, with certain CNVRs becoming fixed in some breeds while others show breed-specific or regional differentiation. In many populations, duplication (gain genotypes) predominates over deletion. Comparative analyses indicate that conserved CNV hotspots are enriched in telomeric and gene-dense chromosomal regions, particularly on chromosome 19, which has approximately 65.58% of its genome covered by CNVRs, compared to chromosome 9 at 27.20% [12].

With regard to the Black Bengal goat, a native Indian breed renowned for its high prolificacy, adaptability, and superior hide quality, this study offers the first comprehensive map of CNVs and CNVRs. Using high-depth whole-genome sequencing, a total of 31,816 CNVs were identified, which were grouped into 8910 CNVRs covering approximately 5.3% of the autosomal genome. The significant predominance of duplications, akin to observations in other livestock species, indicates a natural genomic tendency towards the expansion of gene copies—an evolutionary approach that enhances functional redundancy, allows phenotypic diversity, and increases resilience to environmental shifts.

### 3.1. CNV–CNVR Architecture and Adaptive Evolution

The analysis of genome-wide CNVR distribution showed that the Black Bengal goat genome exhibits a non-random pattern of chromosome distribution. Chromosome 1 was identified as the key CNVR hotspot, with the highest CNVR density and the greatest overlap of CNVs across individuals. Besides Chromosome 1, chromosomes 2, 3, and 4 had more CNVRs than the other chromosomes. However, the number and clustering intensity of CNVRs on these chromosomes were much lower than on Chr1. So, these chromosomes were assigned to the secondary CNVR hotspot region category. This means they had moderate CNVR enrichment rather than strong hotspot formation. The data show that CNVR hotspots are arranged in a hierarchy, with Chr1 as the most important hotspot and Chr2, Chr3, and Chr4 as the next most important levels of structural variation. Targeted structural adaptation is indicated by the dense clusters of genes associated with immunological signaling, extracellular matrix (ECM) architecture, and reproduction seen in these areas. These CNVR-rich segments serve as “evolutionary accelerators,” enabling quick phenotypic adaptations to tropical stresses, and they align with regions of high recombination and segmental duplication. The prevalence of shared CNVRs (~78%) among individuals suggests that both natural and artificial selection have fixed long-standing, breed-stabilized structural variations. Similar CNVR enrichment in adaptive gene clusters has been reported in Ethiopian and Tibetan goats, showing convergent genomic pathways for environmental adaptation [11,13]. The CNVR landscape of the Black Bengal goat thus reflects selection footprints for climate tolerance, prolific reproduction, and dermal integrity—traits that underpin its economic and ecological success in humid subtropical regions [4,10].

### 3.2. Functional Breadth of CNV/CNVR-Associated Genes

Functional annotation of genes overlapping CNVs and CNVRs revealed significant enrichment across four key biological domains—reproduction, immune regulation, metabolism, and extracellular matrix (ECM) organization. Pathway analysis indicated that the main cascades underlying folliculogenesis, tissue remodeling, and immunological resistance include TGF-β signaling, ECM–receptor interaction, and cytokine–cytokine receptor interaction. CNVRs harboring CD80, IL10RB, and USP25 show adaptive amplification of immune-signaling components, suggesting improved parasite resistance and balanced immunoregulation [2,8]. Similarly, under semi-intensive tropical management systems, duplications in the ROBO1 and CADM gene families may contribute to sensory and behavioral adaptation. Taken together, these functionally varied CNVRs provide an example of how structural variations support environmental adaptability and multi-trait resilience in native goat breeds [14,15]. Research into possible genes has demonstrated that CNVs in PPP3CA considerably influence both the quantity and quality of semen in individuals [16]. Several Chinese goat breeds exhibit an association between variations in copy number of KCNJ15, PLEC, and CADM2 and challenges with carcass characteristics, meat quality, and overall growth [17,18]. Selection signature analyses indicate that CNVs have been primary targets of evolution, whether through natural or anthropogenic means. Their contributions encompass the delineation of breed features, such as coat color and reproductive viability [19], as well as fecundity-related parameters, including litter size in Guizhou Black goats [20]. Additionally, structural mutations linked to certain morphological characteristics, such as extra teats, have been effectively found using genome-wide CNV screening, underscoring their functional significance [21].

### 3.3. CNVRs in Reproductive Signaling and Prolificacy

The discovery of CNVRs covering the GDF9 and BMPR1B loci—two major regulators of ovarian folliculogenesis—is noteworthy. Several Black Bengal goats shared these CNVRs, and their high read-depth values (~1.8×) supported duplication events. Both genes regulate granulosa cell proliferation, follicle development, and ovulation rate through their roles in the TGF-β signaling cascade. The well-known FecB (Booroola) mutation in BMPR1B corresponds to a single nucleotide substitution (Q249R) in the coding region, which alters receptor function and is associated with a marked increase in ovulation rate and fecundity in Booroola Merino sheep [22]. The Black Bengal goat’s distinctively large litter size (usually two to three offspring per kidding) may be partly attributed to CNVR-mediated dosage increase at GDF9 and BMPR1B. This supports a gene dosage model of prolificacy, in which structural variations act as quantitative enhancers of fertility in tropical environments [23]. Together, these findings highlight the evolutionary and reproductive significance of CNV-driven modulation of key ovarian genes in influencing prolificacy traits among indigenous goat populations.

### 3.4. CNVRs Shaping Dermal and ECM Architecture

A second group of CNVRs overlapped with collagen and laminin gene families—including COL6A1, COL6A3, LAMC2, LAMB3, FMN1, and CLDN1. These genes encode structural proteins essential for dermal tensile strength, basement-membrane stability, and epithelial cohesion. The breed’s fine-grain, high-tensile skin, which is valued in the leather industry, may be partly supported by duplications in COL6A1 and LAMC2, which improve ECM fiber density and alignment [24]. Hair-follicle sheaths co-express multiple ECM-related CNVRs, suggesting pleiotropic functions in coat formation and dermal strength [25]. As ECM and follicular genes are simultaneously modulated, CNVRs may regulate skin quality, dermal flexibility, and thermoregulation, allowing the breed to adapt to hot, humid areas while maintaining its economic worth.

### 3.5. Integration of Reproductive and Dermal CNVRs

The co-localization of COL6A1/LAMC2/LAMB3 (ECM) and GDF9/BMPR1B (reproduction) CNVRs on adjacent chromosomal regions illustrates a type of adaptive pleiotropy, in which functionally different genes are selected concurrently. This idea of “adaptive reproductive–somatic balance” suggests that CNVs act as genetic mechanisms that balance fertility and somatic resilience in resource-constrained tropical environments. African cattle and goats exhibit integrative selection signals, suggesting convergent genetic adaptation mechanisms to environmental adversity [26,27,28].

### 3.6. Comparative and Evolutionary Context

Compared to cosmopolitan breeds, the Black Bengal goat exhibits greater CNV and CNVR variation. This likely reflects that it has not been artificially selected and has adapted to diverse Indian agro-ecological zones over an extended evolutionary timescale. Unique CNVR signatures associated with GDF9, BMPR1B, and COL6A1 set it apart from commercial breeds like Boer and Saanen [4,24]. The Garole sheep, a well-known Indian breed with BMPR1B-associated mutations correlated with increased ovulation and litter size, demonstrates a comparable genomic pattern [29]. All of these findings show that South Asia is a hub for molecular innovation in the reproductive and adaptive traits of small ruminants. The Black Bengal goat thus exemplifies naturally driven genomic adaptation, balancing prolific reproduction, immune resilience, and dermal robustness within an integrated adaptive framework. Many previous studies used SNP arrays or low-coverage WGS with various computational methods, each with distinct detection sensitivities and biases [30,31]. SNP array-based methods tend to detect larger CNVs and are limited by probe density. Low sequencing depth makes it difficult to identify small CNVs and breakpoints, especially in repetitive regions. In contrast, our study used approximately 10× WGS and a read-depth-based CNVs detection method, which improved the identification of minor and complex CNVs and allowed for more precise boundary definition. These methodological differences likely explain the higher CNV count and better genome coverage observed in Black Bengal goats compared to previous studies in other breeds. Although the small sample size limits conclusions about CNVs prevalence in the broader population, the data remain valuable for building a reliable CNVs database. Comprehensive analyses show that sequencing depth and cohort size have similar impacts on CNV discovery [32].

### 3.7. Breeding and Conservation Implications

The CNVRs characterized here provide valuable genomic resources for marker-assisted breeding and functional selection. For instance, duplications at the GDF9 and BMPR1B loci may be developed into molecular markers for selecting enhanced fecundity animals, whereas CNVRs spanning COL6A1 and LAMC2 could serve as indicators of hide quality. Incorporating CNVR-based information into genomic-selection pipelines has been shown to enhance trait-prediction accuracy in livestock populations [33]. From the standpoint of conservation and breed management, CNVRs act as structural variation fingerprints for native breeds, making it easier to track breed diversity and employ them specifically under national programs like the National Livestock Mission [34].

### 3.8. Limitations and Future Directions

Despite being the first CNV/CNVR atlas for the Black Bengal goat, the discovery of rare variants is constrained by the relatively small sample size (n = 8), which is a common limitation in high-depth whole-genome sequencing-based CNVs studies due to cost and data complexity. Accordingly, the identified CNVs and CNVRs should be interpreted as associative genomic signals rather than causal determinants of phenotypic traits. While several candidate genes were identified within CNVRs, their potential roles in traits such as prolificacy and skin quality remain putative, and direct genotype–phenotype associations were not assessed in the present study. The observed bias toward copy-number gains may reflect both platform-specific detection sensitivities and stringent filtering of deletion events, consistent with previous reports in livestock CNVs studies.

We acknowledge that experimental validation would strengthen these findings; however, due to resource limitations, independent validation (e.g., qPCR) could not be performed in this study. Gene-dosage effects and expression changes should therefore be confirmed through future validation approaches, including qPCR and transcriptomic analyses (e.g., RNA-Seq). Furthermore, integration of CNVR data with GWAS and epigenetic profiles (e.g., DNA methylation) will be essential to better understand the functional relevance of structural variants [2,35]. Future studies involving larger sample sizes and multiple goat populations will be necessary to validate breed-specific CNVs and strengthen inferences regarding their association with economically important traits [36]. Overall, the findings of this study should be considered hypothesis-generating rather than confirmatory, providing a foundation for future integrative and functional genomic investigations.

## 4. Materials and Methods

### 4.1. Sample Collection and DNA Isolation

Blood samples (5 mL) were collected from eight randomly selected Black Bengal goats kept at the Goat Breeding Farm, Jaring, Kalahandi, Odisha, India (Approval vide letter No. 8434/vet.; Dt.13.05.2025). All animal handling and sample collection procedures were conducted in accordance with the guidelines of the Institutional Animal Ethics Committee (IAEC), ICAR–Indian Veterinary Research Institute (ICAR-IVRI), Izatnagar, India. Sampling was performed aseptically using EDTA vacutainers. Genomic DNA was extracted with the QIAGEN DNeasy Blood and Tissue Kit (QIAGEN, Hilden, Germany) according to the manufacturer’s instructions. DNA purity and integrity were assessed using agarose gel electrophoresis, and A260/A280 ratios were determined with a NanoDrop spectrophotometer. Only samples with A260/A280 between 1.7 and 1.9 were used for library preparation.

### 4.2. Library Preparation and Whole Genome Sequencing

Library construction was performed at N2Jenomics Lab Pvt. Ltd., New Delhi, India. All eight samples (Sample IDs: 786, 788, 795, 796, 797, 798, 799, and 801) passed the quality control criteria and were approved for sequencing. Whole-genome sequencing was carried out on the Illumina platform using paired-end chemistry with an average read length of 150 bp. Each library was sequenced to produce approximately 30–35 Gb of clean data per sample, achieving an average coverage of around 10× across the genome. The GC content among samples ranged from 42.7% to 43.5%. The base quality metrics showed that more than 98% of bases had Q20 and more than 94% had Q30 scores, indicating high data quality. For all samples, the mean quality score was above Q36, and over 94% of bases exceeded the Q30 threshold. Data yield per sample ranged from 30–35 GB, ensuring sufficient depth for downstream genome-wide analyses. Pairwise genomic relatedness among individuals was estimated using the genome function in PLINK, which computes identity-by-descent (IBD) coefficients, reported as PI_HAT.

Before alignment, raw sequencing reads underwent quality control and preprocessing to remove adapter sequences and low-quality bases. The reference goat genome assembly (Capra hircus, ARS 1.2) was indexed using BWA, and a sequence dictionary and FASTA index were created with Picard 3.0 and SAMtools 1.23, respectively. High-quality reads were then aligned to the reference genome using the BWA-MEM algorithm. The resulting alignments were converted to BAM format, followed by sorting and indexing with SAMtools. PCR duplicates were identified and removed using Picard tools to minimize potential biases in downstream analyses.

### 4.3. CNV Detection

CNVs were identified using CNVnator v0.4.1 [37], a read-depth-based approach. The genome was partitioned into 100 bp bins, normalized for GC content, and analyzed for deviations from the average read depth. Regions exhibiting read depth ≥1.5× were classified as duplications, whereas those with ≤0.5× were considered deletions, using a significance threshold of *p* < 0.05. CNVs shorter than 1 kb or supported by fewer than 10 reads were excluded to minimize false-positive calls arising from stochastic read-depth fluctuations, particularly under an average sequencing depth of approximately 10×. This read-support threshold ensures sufficient statistical confidence in CNV calls and is consistent with commonly adopted filtering criteria in read-depth-based CNV analyses.

### 4.4. CNVR Construction

To define population-level structural variants, overlapping CNVs across the eight genomes were merged into copy number variation regions (CNVRs) using BEDTools v2.30.0. CNVs separated by ≤1 kb were merged, and each CNVR was assigned a type (duplication or deletion) based on the majority event. Genomic coverage, CNVR density per chromosome, and overlap frequency were calculated using BEDTools.

### 4.5. Functional Annotation and Enrichment

Genes overlapping CNVs or CNVRs (≥50% overlap) were extracted using BEDTools intersect. Functional annotation was performed using PANTHER v17.0 to assign Gene Ontology (GO) terms. Trait-related regions were cross-referenced with the Animal QTLdb (goat) to identify overlaps with fertility, milk, immune, and skin-related QTLs.

### 4.6. Statistical and Visualization Analyses

CNVs were classified by size (<1–10 kb, 10–100 kb, 100 kb–1 Mb, >1 Mb). Lengths were log10-transformed, and kernel density plots were generated to show CNV size distributions. Duplications and deletions per sample were compared using grouped bar charts, while CNVs and CNVR densities per chromosome were displayed as normalized bar plots. CNVR sharing frequencies across individuals were summarized with stacked bar plots and heatmaps. For annotation we have utilized BEDTools (v2.30.0). All genomic coordinates were based on the *Capra hircus* ARS 1.2 assembly (GCA_001704415.2).

## 5. Conclusions

Using whole-genome sequencing of eight individuals, this study presents the first high-resolution genome-wide CNVs and CNVR atlas for the Black Bengal goat in India. CNVR hotspots were primarily found on chromosomes 1 and contained functionally relevant regions enriched for genes linked to metabolism, immune function, extracellular matrix (ECM) organization, and reproduction. Notably, the breed’s high prolificacy may be probably supported by recurrent duplications spanning GDF9 and BMPR1B within the TGF-β signaling pathway, while CNVRs involving COL6A1, LAMC2, LAMB3, FMN1, and CLDN1 offer a structural-genomic explanation for the superior skin quality and strength.

Taken together, these duplication events highlight the potential role of CNVR-driven gene dosage effects in contributing to the economic and adaptive traits of Black Bengal goats; however, these associations are preliminary and warrant further functional and phenotypic validation. The CNVRs identified in this study are important genomic resources for conservation initiatives, genomic prediction, and marker-assisted selection. The effectiveness of multi-trait selection, especially for fecundity and hide quality, may be enhanced by incorporating CNVR-based markers in breeding pipelines. To establish direct linkages between genotype and phenotype, future research should incorporate integrated multi-omics analyses, population-scale association studies, and functional validation (e.g., qPCR-based CNVs quantification). In conclusion, CNVR-mediated structural variation may play an important role in the Black Bengal goat’s capacity for both adaptive evolution and production. In addition to improving our knowledge of structural genomics in small ruminants, this foundational CNVR map establishes the framework for precision breeding and the long-term preservation of India’s native genetic resources.

## Figures and Tables

**Table 1:** CNV Summary per Sample.

Sample	Total CNVs	Total Length (bp)	Mean Length (bp)	Median Length (bp)	Max Length (bp)	Min Length (bp)	% Genome Coverage
786	857	3.89 × 10^7^	45,453	36,102	2.8 × 10^6^	1124	1.3%
795	4056	1.99 × 10^8^	49,203	40,900	1.8 × 10^6^	1078	6.6%
796	7230	3.42 × 10^8^	47,295	40,870	2.4 × 10^6^	1122	11.4%
797	4100	1.96 × 10^8^	47,879	39,667	1.9 × 10^6^	1120	6.5%
798	3680	1.66 × 10^8^	45,534	38,214	2.2 × 10^6^	1140	5.5%
799	3840	1.79 × 10^8^	46,685	39,090	2.1 × 10^6^	1129	6.0%
801	2830	1.42 × 10^8^	49,076	41,535	2.5 × 10^6^	1134	4.7%
788	290	1.23 × 10^7^	42,569	38,899	2.36 × 10^5^	2899	0.41%

**Table 2:** Top 20 Recurrent CNV-Overlapping Genes Across Eight Individuals.

Gene Symbol	Chr	Genomic Position (Mb)	No. of Samples	Functional Category
LOC108636892	Chr10	78.441–78.442	8	Uncharacterized/possible regulatory locus
LOC102178529	Chr7	93.063–93.111	8	Non-coding RNA/transcriptional regulator
LOC108638022	Chr18	60.227–60.243	8	Regulatory pseudogene
LRRTM4	Chr11	58.220–59.227	8	Synaptic signaling and axonal guidance
LOC108636750	Chr9	88.021–88.052	8	Hypothetical gene with potential structural role
ROBO1	Chr1	24.804–25.920	7	Axon guidance and cell adhesion signaling
GBE1	Chr1	27.824–28.134	7	Glycogen metabolism and energy adaptation
TIAM1	Chr1	2.425–2.887	7	Cell migration and signal transduction
COL6A1	Chr1	145.631–145.652	6	Collagen fibril assembly and dermal structure
LAMC2	Chr16	62.840–62.910	5	Basement membrane adhesion
LAMB3	Chr16	72.302–72.344	5	ECM integrity and skin resilience
CD80	Chr1	64.007–64.035	4	Immune activation and cytokine signaling
IL10RB	Chr1	0.725–0.756	4	Cytokine receptor subunit for immune modulation
USP25	Chr1	19.763–19.933	4	Protein deubiquitination and immune homeostasis
BMPR1B	Chr6	29.894–30.342	3	Ovarian follicular development and BMP signaling
GDF9	Chr7	66.023–66.028	3	Oocyte development and follicular growth (BMP signaling)
CLDN1	Chr1	76.761–76.779	3	Tight junction and epithelial barrier function
FMN1	Chr10	73.144–73.581	3	Cytoskeletal organization and cell morphogenesis
NXPE3	Chr1	45.853–45.913	2	Cell adhesion and protein interaction (Ig-like domain)
KITLG	Chr5	18.044–18.151	2	Growth factor signaling and cell proliferation

**Table 3:** Key CNVR-Associated Functional Genes Identified in High-Confidence Datasets.

**Gene Name**	**Chromosome**	**Start (Mb)**	**End (Mb)**	**CNV Type**	**Approx. Size (bp)**	**Function**
GDF9	7	66.024	66.029	Duplication	4720	Folliculogenesis, oocyte maturation
BMPR1B	6	29.895	30.343	Duplication	448,166	TGF-β signaling, ovulation rate
COL6A1	1	145.631	145.652	Duplication	21,131	Collagen assembly, ECM structure
LAMC2	16	62.841	62.91	Duplication	69,921	Basement membrane anchoring
LAMB3	16	72.302	72.344	Duplication	42,506	ECM adhesion, dermal cohesion
FMN1	10	73.144	73.582	Duplication	437,404	Dermal cytoskeleton organization
CD80	1	64.007	64.035	Duplication	28,018	Immune activation
IL10RB	1	0.725	0.756	Duplication	30,520	Cytokine receptor signaling
ROBO1	1	24.804	25.92	Duplication	1,116,119	ECM signaling, immunity
USP25	1	19.764	19.934	Duplication	170,232	Ubiquitin pathway, inflammation
GBE1	1	27.824	28.135	Duplication	310,726	Glycogen metabolism
TIAM1	1	2.425	2.888	Duplication	462,470	Cell signaling, metabolic adaptation
NXPE3	1	45.853	45.913	Duplication	59,667	Metabolic resilience and energy regulation

## Data Availability

The data presented in this study are available on request from the corresponding author. The WGS dataset for the 8 Black Bengal Goats used in this study is with the corresponding author. The sequencing data generated in this study are part of an ongoing Government of India–funded program under the National Livestock Mission (NLM) project and are therefore subject to institutional and governmental data-sharing policies. At present, these datasets are considered restricted and cannot be deposited in a public repository, such as NCBI SRA, without prior approval from the relevant authorities.

## Supplementary Materials

The following supporting information can be downloaded at: https://www.mdpi.com/article/10.3390/ijms27094045/s1.
