# Supplementary material for: Insights into Copy Number Variation Architecture in Black Bengal Goat Genome
Source: Int J Mol Sci. 2026 Apr 30;27(9):4045. doi: 10.3390/ijms27094045 (PMC13163300; doi:10.3390/ijms27094045)
Supplement: Supplementary file 1 [file ijms-27-04045-s001.zip › Suppl Table S1.pdf]

Chromosome	Start (bp)	End (bp)	CNV Type	Representative Gene(s)	Functional Relevance
Chr2	~22.4 Mb	~22.5 Mb	Duplication	**LAMC2, LAMB3**	Extracellular matrix, tissue integrity
Chr6	~12.42 Mb	~12.48 Mb	CNVR hotspot	**BMPR1B**	Fecundity, ovulation rate
Chr6	~24.90 Mb	~24.94 Mb	CNVR hotspot	**GDF9**	Oocyte development, fertility
Chr1	40.8–41.0 Mb	~41 Mb	Duplication	LOC genes (cluster)	CNV hotspot region
Chr1	44.9–45.2 Mb	~45 Mb	Duplication	ABI3BP	Cell adhesion, tissue remodelling
Chr1	57–59 Mb	~59 Mb	Duplication	SLC9C1, ZBTB20	Metabolic regulation
Chr1	76–77 Mb	~77 Mb	Duplication	P3H2	Collagen modification

Supplementary Table S1. CNVR-enriched regions and associated genes in Black Bengal goat
